# Evaluation of high dose post-dialytic versus daily beta-lactam dosing in hemodialysis patients using Monte Carlo simulation

**DOI:** 10.1007/s11096-025-02044-5

**Published:** 2025-11-26

**Authors:** Andrew J. Bauman, Olivia G. Caiazza, Nerissa Wan, Sydnee Payer, Olivia G. Pauly, Sierra Shoemaker, Susan J. Lewis

**Affiliations:** 1 College of Pharmacy, University of Findlay, Findlay, OH USA; 2 Department of Pharmacy, University Hospitals, Warrensville Heights, OH USA; 3 Department of Pharmacy, HonorHealth Sonoran Crossing Medical Center, Phoenix, AZ USA; 4 Department of Pharmacy, Cleveland Clinic – Hillcrest Hospital, Mayfield Heights, OH USA; 5 Department of Pharmacy Practice, College of Pharmacy, University of Findlay, Findlay, OH USA; 6 Department of Pharmacy, Mercy Health - St. Anne Hospital, Toledo, OH USA

**Keywords:** Cefepime, Ceftazidime/avibactam, Hemodialysis, Meropenem, Monte Carlo simulation, Pharmacokinetics/pharmacodynamics, Post-HD dosing

## Abstract

**Introduction:**

Daily intravenous dosing of cefepime, meropenem and ceftazidime/avibactam is recommended in patients receiving intermittent hemodialysis (IHD), but requires hospitalization or frequent clinic visits. High dose post-HD administration may offer a more convenient outpatient alternative, but supporting data is limited.

**Aim:**

To evaluate the feasibility by assessing predicted pharmacokinetic/pharmacodynamic (PK/PD) target attainment and neurotoxicity risk of high-dose post-HD versus daily dosing strategies for cefepime, meropenem, and ceftazidime/avibactam in patients receiving thrice-weekly IHD, using the Monte Carlo simulation (MCS) techniques.

**Method:**

One-compartment pharmacokinetic models were developed using published data to simulate drug exposure in anuric patients receiving 4-h IHD thrice-weekly. MCS (Crystal Ball, Oracle) assessed the probability of target attainment (PTA) and neurotoxicity risk of various post-HD and daily dosing regimens in 5,000 virtual cohorts for one week. The PK/PD targets were ≥ 60% *f*T > MIC for cefepime, ≥ 40% *f*T > MIC for meropenem and ≥ 50% *f*T > MIC for ceftazidime with ≥ 50% fT > 1 g/mL for avibactam, assuming *Pseudomonas aeruginosa* or *Enterobactarales* infections. A PTA ≥ 90% was considered optimal for PK/PD target attainment. Safety was also assessed using the neurotoxicity thresholds.

**Results:**

All daily regimens achieved PTA ≥ 90% on all simulated days. High-dose post-HD cefepime (1–2 g) and meropenem (2 g) maintained acceptable PTA over 2-day interdialytic periods, but failed to sustain targets through the 3-day period. Ceftazidime/avibactam post-HD dosing (0.94 g–0.94 g–2.5 g) maintained ≥ 90% PTA for ceftazidime throughout the week, though avibactam fell slightly below target on the final day. Predicted neurotoxicity risk was negligible for meropenem and ceftazidime/avibactam, but elevated with higher cefepime doses (1–2 g post-HD and 1 g daily). Cefepime 0.5 g daily, meropenem 0.25–1 g daily, and ceftazidime/avibactam 0.94 g daily or 0.94 g–0.94 g–2.5 g post-HD attained both PK/PD targets and safety targets.

**Conclusion:**

High-dose post-HD dosing appears feasible for ceftazidime/avibactam but may be inadequate for cefepime and meropenem over a 3-day interdialytic period. Elevated neurotoxicity risk predicted with higher cefepime doses highlights the importance of cautious dosing and consideration of therapeutic drug monitoring.

**Supplementary Information:**

The online version contains supplementary material available at 10.1007/s11096-025-02044-5.

## Impact statements

- This study evaluated the feasibility of high-dose post-HD dosing of cefepime, meropenem, and ceftazidime/avibactam in patients with kidney failure on thrice-weekly hemodialysis, using Monte Carlo simulation (MCS) as a potential outpatient alternative to daily dosing.
- MCS predicted that post-HD cefepime and meropenem dosing may be feasible for 2-day interdialytic intervals but not the 3-day interval, whereas ceftazidime/avibactam appeared feasible for both.
- Clinicians should avoid high-dose cefepime post-HD regimens across 3-day interdialytic periods due to elevated neurotoxicity risk and consider TDM where available.

## Introduction

Infectious complications are a leading cause of increased morbidity and mortality in patients with end stage kidney disease receiving intermittent hemodialysis (IHD), largely due to the need of vascular access and underlying immunodeficiency [1]. These patients are particularly vulnerable to infections caused by resistant organisms, including multidrug-resistant Gram-negative bacteria, requiring the use of broad spectrum or novel intravenous (IV) antibiotics such as cefepime, meropenem or ceftazidime/avibactam [2]. These β-lactam antibiotics are primarily excreted by the kidney and have relatively short half-lives (1–3 h) in individuals with normal kidney function. However, in anuric patients undergoing IHD, drug clearance is markedly prolonged with reported half-lives of ~ 13.5 h for cefepime, 7–9.3 h for meropenem, 25.3 h for ceftazidime, and 22.8 h for avibactam [3–7], raising concern for drug accumulation and associated toxicity, particularly neurotoxicity. Although β-lactam antibiotics are generally well-tolerated, elevated plasma concentrations have been linked with neurotoxicity in retrospective studies and case reports [8–12]. Current recommendations for patients with a typical thrice-weekly IHD are daily dosing for these agents, to maintain therapeutic drug levels [13–15]. However, daily dosing necessitates hospitalization or daily clinic visits, posing logistical burdens and negatively affecting patient quality of life.

To address this, post-HD dosing strategies using higher doses have been proposed as a more convenient outpatient alternative, yet supporting pharmacokinetic (PK) and clinical data remain limited. Two small cefepime PK studies suggested that 1–2 g post-HD dosing may maintain therapeutic concentrations for 2–3-day interdialytic intervals, and a small outcome study reported favorable clinical responses with 2 g post-HD dosing [16–18]. For meropenem, one small retrospective study described post-dialytic administration as a promising approach but did not provide standardized dosing recommendations [19]. While these limited findings suggest that post-HD dosing may be feasible for cefepime and meropenem, important knowledge gaps remain regarding optimal dosing regimens that ensure both effectiveness and safety. In addition, no PK or clinical studies have evaluated ceftazidime/avibactam in patients receiving IHD, warranting further investigation into its feasibility and optimal post-HD dosing in this population. A recent review likewise highlighted the paucity of evidence to guide standardized post-HD antimicrobial dosing in patients with kidney failure receiving IHD [20]. Modeling and simulation approaches can help address this gap by evaluating how different dosing schedules relative to IHD influence drug exposure [21–23] and by informing clinical dosing decisions.

### Aim

This study aimed to assess the feasibility of high-dose, post-HD cefepime, meropenem, and ceftazidime/avibactam dosing compared with conventional daily dosing strategies in patients receiving thrice-weekly IHD, by evaluating predicted pharmacokinetic/pharmacodynamic (PK/PD) target attainment and risk of neurotoxicity, using the Monte Carlo simulation (MCS) techniques.

## Method

### Pharmacokinetic model implementation

One-compartment, first-order PK models were constructed in Microsoft Excel using pertinent demographic and PK data to generate the free plasma concentration profiles of cefepime, meropenem and ceftazidime/avibactam in patients with kidney failure undergoing chronic IHD for 4 h, thrice-weekly (Tue-Thu-Sun). All simulated patients were assumed to be anuric with negligible or no residual kidney function. Model input parameters are outlined in Table 1. Body weight, non-renal clearance (CL_NR_), volume of distribution (Vd), and protein binding were derived from published literature [3, 4, 6, 7, 13–15, 23–29]. Body weight was included as a fixed input rather than a covariate. Protein binding was used to estimate free plasma concentrations. Regression analyses of published transmembrane clearance at various effluent flow rates were performed to find best-fit relationships and to extrapolate drug specific saturation coefficients (SA) at the desired dialysate flow rate (Qd) [3, 6, 7, 22, 24–39]. A variability of 20% was applied to SA values to simulate interpatient variability. Due to scarce PK data on avibactam, its transmembrane clearance (8.97 L/hr) was adopted from a single IHD study using a Qd of 500 mL/min [7], rather than estimated from SA. High flux IHD typically uses Qd of 600–800 mL/min in the U.S., therefore, 800 mL/min was applied for cefepime and meropenem. For ceftazidime/avibactam, Qd of 500 mL/min was used to align with the avibactam reference study conducted in Europe [7]. All input data was assumed to follow a log-Gaussian distribution. The following equations were used in the model:

$${\mathrm{CL}}_{{{\mathrm{IHD}}}} = {\mathrm{SA}} \times {\mathrm{Qd}}$$

$$Ke\__{{{\mathrm{on}} - {\mathrm{IHD}}}} = \, \left( {{\mathrm{CL}}_{{{\mathrm{NR}}}} + {\mathrm{CL}}_{{{\mathrm{IHD}}}} } \right) \, /{\text{ Vd}}$$

$$Ke\__{{{\mathrm{off}} - {\mathrm{IHD}}}} = {\text{ CL}}_{{{\mathrm{NR}}}} /{\text{ Vd}}$$

where CL_IHD_ = transmembrane drug clearance during dialysis, SA = saturation coefficient, Qd = dialysate flow rate, CL_NR_ = non-renal clearance, Vd = volume of distribution, *Ke*__on-IHD_ = elimination rate constant during dialysis, *Ke*__off-IHD_ = elimination rate constant during the interdialytic period.

**Table 1:** Demographic & Pharmacokinetic Input Parameters [3, 4, 6, 7, 22, 24–39]

Input parameters	Cefepime	Meropenem	Ceftazidime	Avibactam
Weight (kg)	93.5 ± 30	93.5 ± 30	93.5 ± 30	93.5 ± 30
Volume of Distribution (L/kg)	0.24 ± 0.07	0.24 ± 0.05	0.236 ± 0.03	0.28 ± 0.15
Non-Renal Clearance (mL/min)	11.6 ± 1.15	25 ± 6	10.6	13.3 ± 2.5
Free Fraction	0.79 ± 0.09	0.98	0.83 ± 0.07	0.93 ± 0.01
Saturation Coefficient	0.17 ± 0.03	0.13 ± 0.03	0.22 ± 0.04	N/A

All values are expressed as mean ± SD

### Pharmacodynamic and safety targets

Β-lactams exhibit time-dependent bactericidal effect and its effectiveness is related to the time that free plasma drug concentrations exceed above the minimum inhibitory concentration of a causative pathogen (*f*T > MIC) [40, 41]. No studies have defined optimal PK/PD targets associated with clinical outcomes in IHD patients. Thus, we used the generally accepted β-lactam PK/PD targets, assuming patients are not critically ill: ≥ 60%*f*T > MIC for cefepime, ≥ 50%*f*T > MIC for ceftazidime, and ≥ 40%*f*T > MIC for meropenem [40, 41]. For avibactam, the target was defined as attaining free plasma concentrations above a threshold concentration (C_T_) of 1 mg/L for at least 50% of the dosing interval (≥ 50%*f*T > C_T_) [42]. The simulation modeled *Pseudomonas aeruginosa* as the reference pathogen for cefepime, and *Enterobacterale* for meropenem and ceftazidime/avibactam. Susceptibility breakpoint MICs from the Clinical and Laboratory Standards Institute (CLSI) were used: 8 mg/L for cefepime against *P. aeruginosa*, and 2 mg/L and 8 mg/L for meropenem and ceftazidime, respectively, against *Enterobacteriaceae* [43].

**Fig. 1:**
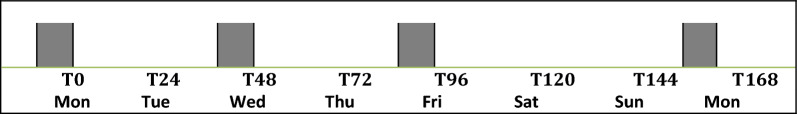
Simulated Intermittent Hemodialysis and β-lactam Dosing Schedules Grey boxes represent 4-h IHD sessions (Tue/Thu/Sun). Daily regimens were administered every 24 h starting at T0; post-HD regimens were administered immediately after each HD session (T0, T48, T96). Toxicity risk was evaluated at the end of each simulated day (T24, T48, T72, etc.)

The total trough plasma concentrations of 20 mg/L and 64 mg/L were suggested to increase the risk of neurotoxicity for cefepime and meropenem, respectively [8–10]. Data on ceftazidime with or without avibactam is limited regarding neurotoxicity, but it is generally recommended to avoid trough concentrations exceeding 80 mg/L to minimize the risk of toxicity [44, 45]. Hence, the risk of neurotoxicity for each study agent was evaluated using these suggested toxicity thresholds (20 mg/L for cefepime, 64 mg/L for meropenem, and 80 mg/L for ceftazidime) at the end of each simulated day. In our model, these end of day concentrations corresponded to the trough within each dosing interval (i.e., immediately prior to the next daily dose or at the end of HD session before the subsequent post-HD dose) as depicted in Fig. 1 The neurotoxicity risk was calculated as the proportion of simulated patients whose concentrations exceeded the respective toxicity thresholds at these time points.

### Monte carlo simulation

The probability of target attainment (PTA) of various daily and post-HD dosing strategies were assessed using MCS (Crystal Ball Classroom Edition, Oracle). Free plasma drug concentration–time profiles were generated over a one-week treatment period in 5,000 virtual patients for each dosing scenario. Simulated dosing regimens included: 1) Cefepime 0.5 g daily, 1 g daily, 1 g post-HD, and 2 g post-HD; 2) meropenem 0.25 g daily, 0.5 g daily, 1 g daily, 0.5 g post-HD, 1 g post-HD, and 2 g post-HD and 3) ceftazidime/avibactam (administered in a fixed 4:1 ratio; e.g., the 0.94 g dose consist of ceftazidime 0.75 g and avibactam 0.19 g): 0.94 g daily, 0.94 g post-HD, 0.94 g-1.5 g-1.5 g post-HD, and 0.94 g-0.94 g-2.5 g post-HD. Infusion durations were modeled as 30 min for cefepime and meropenem, and 2 h for ceftazidime/avibactam reflecting standard administration practices [13–15]. For daily regimens, doses were administered every 24 h and scheduled after completion of the IHD session on dialysis days (Fig. 1). An effective dosing regimen was defined as one achieving ~ 90% PTA on each day throughout the simulated treatment week.

### Ethics approval

This is a simulation study using published data. Thus, no ethical approval is required.

## Results

**Fig. 2:**
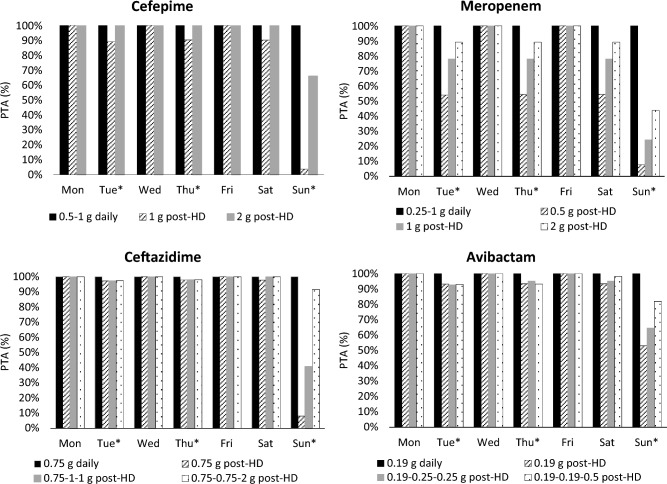
Probability of Target Attainment (PTA) for Cefepime, Meropenem, Ceftazidime and Avibactam Regimens in Patients Receiving Thrice-Weekly Hemodialysis Over a One-Week Interval Each panel displays the PTA (%) over a one-week treatment period for cefepime, meropenem, ceftazidime, and avibactam, comparing daily and post-hemodialysis (HD) dosing regimens. For cefepime and meropenem, all simulated daily dosing regimens achieved 99–100% PTA and are represented by a single blue bar. Each bar represents PTA on consecutive days (Mon–Sun) in 5,000 virtual anuric patients undergoing 4-h hemodialysis sessions on Tuesday, Thursday, and Sunday (marked with an asterisk). Pharmacodynamic targets were defined as: ≥ 60% fT > MIC (8 mg/L) for cefepime, ≥ 40% fT > MIC (2 mg/L) for meropenem, ≥ 50% fT > MIC (8 mg/L) for ceftazidime, and ≥ 50% fT > 1 mg/L for avibactam. A PTA ≥ 90% was considered optimal for target attainment

The PTA results for cefepime, meropenem, and ceftazidime/avibactam daily and post-HD dosing regimens in virtual patients receiving thrice-weekly IHD are depicted in Fig. 2 Detailed PTA values for each simulated dosing regimen are provided in the supplementary material.

### Cefepime

Daily cefepime dosing (500 mg and 1 g) consistently achieved PTA 99–100% on both HD and non-HD days for the PK/PD target of ≥ 60% fT > MIC. Post-HD dosing with 1 g and 2 g maintained PTA ≥ 90% on most days, but PTA fell below 90% on the final day of the 3-day interdialytic period, resulting in 3.7% and 65.9% with the 1 g and 2 g doses, respectively.

### Meropenem

All simulated meropenem daily regimens (0.25 g, 0.5 g, and 1 g) attained 99–100% PTA on every treatment day for the PK/PD target of ≥ 40% fT > MIC. Post-HD regimens (0.5 g, 1 g, and 2 g) achieved ≥ 90% PTA on the day of antibiotic administration but dropped below 90% on subsequent days. Notably, the 2 g post-HD regimen achieved a PTA of 89.2% on the second day and 43.8% on the third day of the 3-day interdialytic period.

### Ceftazidime/avibactam

For ceftazidime, the 0.75 g daily dosing regimen maintained PTA 100% across all days of the treatment week for the PK/PD target of ≥ 50% fT > MIC. Among the ceftazidime post-HD regimens, both 0.75 g-0.75 g-0.75 g and 0.75 g-1 g-1 g dosing failed to sustain PTA ≥ 90% on the third day of the 3-day interdialytic period. However, the 0.75 g-0.75 g-2 g post-HD dosing regimen achieved PTA ≥ 90% on all days. Avibactam resulted in a similar pattern for the ≥ 50% fT > CT target; however, the 0.19 g-0.19 g-0.5 g regimen, accompanied with ceftazidime 0.75 g-0.75 g-2 g, did not maintain PTA ≥ 90% on the third day of the 3-day interdialytic period (81.9%).

### Risk of toxicity

The potential for drug-induced neurotoxicity was also evaluated based on the suggested total plasma concentration thresholds. No risk was predicted for any meropenem or ceftazidime/avibactam regimens, with all probabilities of risk remaining at 0%. In contrast, MCS findings showed a notable risk of exceeding the toxicity threshold with cefepime, depending on the dosing strategy shown in Table 2. The 0.5 g daily regimen resulted in negligible risk (0–2.1%) across all days. However, the 1 g daily regimen increased the predicted risk to 71–99.7% on non-dialysis days and 0–42% on dialysis days in 5,000 virtual patients. Post-HD dosing regimens also elevated toxicity risk on non-dialysis days, with 70.3–86.6% and 48.8–100% of virtual patients exceeding the threshold following 1 g and 2 g post-HD dosing respectively.

**Table 2:** Probability of increased neurotoxicity of daily vs. post-HD cefepime dosing regimens in patients receiving thrice-weekly hemodialysis

Dose	Mon	Tue*	Wed	Thu*	Fri	Sat	Sun*
0.5 g daily	0%	0%	0%	0%	0%	2.1%	0%
1 g daily	71.0%	0%	95.6%	0%	95.6%	99.7%	42.0%
1 g post-HD	70.3%	0%	86.6%	0%	86.0%	0%	0%
2 g post-HD	100%	0%	100%	0%	100%	48.8%	0%

^*^Gray columns indicate then days when hemodialysis is scheduled; Neurotoxicity risk was assessed using the simulated plasma total concentrations exceeding the toxicity threshold ≥ 20 mg/L at the end of each day

## Discussion

High dose post-HD antibiotic administration presents a practical outpatient alternative to daily IV therapy in patients undergoing IHD, reducing the need for hospitalization or daily clinic visits. However, its current use is mainly limited to a few agents such as vancomycin, daptomycin, cefazolin, and aminoglycosides, due to limited PK/PD data supporting broader application. To our knowledge, this is the first study to use MCS to evaluate both PK/PD target attainment and potential neurotoxicity risk for high-dose, post-HD dosing regimens of cefepime, meropenem, and ceftazidime/avibactam, compared with conventional daily dosing regimens in virtual IHD patient cohorts.

Our MCS analysis suggests that while conventional daily regimens of all three antibiotics maintain adequate PK/PD target attainment throughout the treatment week, high dose post-HD dosing strategies yield more variable results. For cefepime and meropenem, even the highest conventional post-HD dose (2 g) was insufficient to maintain PK/PD target attainment over the 3-day interdialytic period. Although higher post-HD doses may be required to maintain PK/PD targets during the 72-h interdialytic period, exceeding currently approved maximum doses would raise safety concerns and should be carefully weighed in future clinical evaluations. In contrast, for ceftazidime/avibactam, a stepped post-HD dosing strategy (0.94 g-0.94 g-2.5 g) was predicted to sustain PK/PD target of ceftazidime throughout the week, although avibactam exposure fell slightly below the 90% PTA threshold on the final day of 3-day interdialytic period. Although post-HD ceftazidime/avibactam dosing appears pharmacokinetically feasible, the additional 2-h infusion following a 4-h IHD session may limit acceptability. However, this strategy may still be useful in select cases, such as when daily outpatient infusions are not feasible or hospitalization would otherwise be required. Therefore, feasibility and acceptability will depend on patient circumstances and dialysis center resources.

As aforementioned, available data on post-HD dosing strategies for cefepime and meropenem are limited to a few small studies, and no studies have evaluated ceftazidime/avibactam in IHD patients. Schmaldienst et al. (n = 6) reported that a 2 g post-HD cefepime dose in patients undergoing 3.5-h IHD (Qd = 500 mL/min) was sufficient to maintain the target MIC (8 mg/L) during 2–3 day interdialytic periods [16], with an 82% clinical success rate and only mild adverse events [17]. Descombes et al. (n = 9) assessed an individualized post-HD cefepime dosing (15 mg/kg; 750–1500 mg) in patients on 4-h IHD (Qd = 500 mL/min) [18]. Mean pre-HD concentrations (~ 11 mg/L) over 2–3 day interval exceeded the MICs for *Enterobacterales* (1 mg/L) but not consistently for *P. aeruginosa* (8 mg/L), leading to a recommendation of 1–1.5 g for the 2–3 day interdialytic periods, or up to 2 g with therapeutic drug monitoring (TDM) for resistant pathogens or residual renal function. In contrast, our MCS findings suggest that 1–2 g post-HD cefepime is unlikely to maintain PK/PD targets over the 3-day interdialytic period in anuric patients. This discrepancy may be due to longer HD duration, faster Qd, and larger simulated body weights (~ 93.5 kg vs. 62–63 kg), resulting in higher dialytic clearance and reduced exposure. Data on post-HD meropenem are even more limited. One retrospective study comparing daily and post-HD regimens showed shorter hospital stays with post-HD dosing, but higher 30-day readmission rates [19]. However, the lack of PK data and standardized dosing limits meaningful comparison with our findings.

A key concern with high-dose post-HD β-lactam dosing is the potential for drug-induced neurotoxicity. In this study, none of the tested meropenem or ceftazidime/avibactam dosing regimens, whether post-HD or daily, resulted in drug accumulation or predicted toxicity. Conversely, cefepime post-HD regimens (1 g and 2 g) as well as the 1 g daily regimen resulted in a substantially elevated risk of neurotoxicity, particularly on non-dialysis days. Notably, 0.5 g daily cefepime was the only regimen that achieved both PK/PD and safety targets. These findings suggest that high-dose post-HD dosing is not more toxic than daily dosing; rather, both dosing approaches can pose a risk, if not appropriately dosed. This risk likely stems from the narrow margin between cefepime’s PK/PD target predicted free plasma concentrations (≥ 8 mg/L) and the neurotoxicity threshold (predicted total plasma concentrations of ≥ 20 mg, assuming 20% protein binding). By comparison, the PK/PD target concentrations for meropenem (≥ 2 mg/L) and ceftazidime (≥ 8 mg/L) are well below their respective toxicity thresholds (64 mg/L and 80 mg/L), resulting in a lower predicted risk of neurotoxicity.

Cefepime-induced neurotoxicity is well-documented, particularly in patients with renal impairment or critical illness [46, 47]. Although reports in patients experiencing this are relatively rare, at least one case described neurotoxicity following cefepime 1 g every 12 h for five days which is significantly higher than those typically recommended in IHD patients [47]. Despite cefepime’s high dialyzability (70–85%), substantial drug accumulation may occur in anuric patients during prolonged interdialytic periods, increasing the risk of toxicity**.** Our study further underscores the importance of cautious dosing in this patient population. When feasible, TDM should be performed to guide dosing and minimize risk of neurotoxicity.

This study has several limitations inherent to PK modeling and simulation. First, our virtual patient cohort was constructed using published PK parameters and body weight data from adult patients undergoing thrice-weekly IHD, assuming anuria, uninterrupted dialysis, and fixed Qd (800 mL/min for cefepime and meropenem; 500 ml/min for ceftazidime/avibactam). Therefore, the findings are most applicable to patients with similar clinical characteristics. Factors such as residual kidney function, different dialysis parameters, or patient-specific variables (e.g. infusion duration, serum albumin levels, fluid status) that may influence drug disposition were not incorporated. Secondly, our model used generally accepted PK/PD targets (≥ 60%*f*T > MIC for cefepime, ≥ 50%*f*T > MIC for ceftazidime, and ≥ 40%*f*T > MIC for meropenem) with CLSI breakpoints, assuming clinically stable outpatients. However, infections caused by resistant pathogens may require higher exposure [44]. Lastly, neurotoxicity threshold for cefepime was based on limited retrospective data and may not fully capture interpatient variability. While our findings highlight the elevated neurotoxicity risk with certain regimens, individualized assessment and clinical validation remain essential to guide dosing and minimize toxicity.

## Conclusion

MCS enabled evaluation of PK/PD target attainment and probability of neurotoxicity risk of post-HD vs. daily dosing strategies for cefepime, meropenem, and ceftazidime/avibactam in patients receiving thrice-weekly IHD. While post-HD dosing of ceftazidime/avibactam appear feasible, conventional high-dose post-HD administration of cefepime and meropenem are likely insufficient to sustain target exposures over the 3-day interdialytic period, suggesting a need for higher doses or redosing on the final day. Conversely, cefepime 0.5 g and meropenem 0.25 g –1 g daily regimens achieved both PK/PD targets and safety targets. Higher cefepime doses, whether administered daily or post-HD, were predicted to elevate neurotoxicity risk and warrant clinician caution, vigilant monitoring, and consideration of TDM. Clinical studies are needed to validate these MCS findings.

## Supplementary Information

Below is the link to the electronic supplementary material.

Supplementary file1 (DOCX 15 kb)

## Data Availability

The datasets generated during the current study are available from the corresponding author upon reasonable request.
