# Supplementary material for: Evaluation of high dose post-dialytic versus daily beta-lactam dosing in hemodialysis patients using Monte Carlo simulation
Source: Int J Clin Pharm. 2025 Nov 26;48(2):636–43. doi: 10.1007/s11096-025-02044-5 (PMC12992419; doi:10.1007/s11096-025-02044-5)
Supplement: Supplementary file 1 — Supplementary file1 (DOCX 15 kb) [file 11096_2025_2044_MOESM1_ESM.docx]

Table. Probability of target attainment of daily vs. post-HD cefepime, meropenem, and ceftazidime/avibactam regimens in patients receiving thrice-weekly hemodialysis

| ***Cefepime*** | | | | | | | |
| --- | --- | --- | --- | --- | --- | --- | --- |
| **Dose** | **Mon** | **Tue** | **Wed** | **Thu** | **Fri** | **Sat** | **Sun** |
| 0.5 g daily | **99.7%** | **100%** | **100%** | **100%** | **100%** | **100%** | **100%** |
| 1 g daily | 100% | 100% | 100% | 100% | 100% | 100% | 100% |
| 1 g post-HD | 100% | 89.0% | 100% | 90.3% | 100% | 90.2% | 3.7% |
| 2 g post-HD | 100% | 99.9% | 100% | 99.9% | 100% | 99.9% | 65.9% |
| ***Meropenem*** | | | | | | | |
| **Dose** | **Mon** | **Tue** | **Wed** | **Thu** | **Fri** | **Sat** | **Sun** |
| 0.25 g daily | **99.8%** | **99.8%** | **99.8%** | **99.8%** | **99.8%** | **99.8%** | **99.8%** |
| 0.5 g daily | **100%** | **100%** | **100%** | **100%** | **100%** | **100%** | **100%** |
| 1 g daily | **100%** | **100%** | **100%** | **100%** | **100%** | **100%** | **100%** |
| 0.5 g post-HD | 100% | 54.0% | 100% | 54.5% | 100% | 54.5% | 7.7% |
| 1 g post-HD | 100% | 77.9% | 100% | 77.9% | 100% | 77.9% | 24.3% |
| 2 g post-HD | 100% | 89.2% | 100% | 89.2% | 100% | 89.2% | 43.8% |
| ***Ceftazidime*** | | | | | | | |
| **Dose** | **Mon** | **Tue** | **Wed** | **Thu** | **Fri** | **Sat** | **Sun** |
| 0.75 g daily | **100%** | **100%** | **100%** | **100%** | **100%** | **100%** | **100%** |
| 0.75 g post-HD | 100% | 97.2% | 100% | 97.8% | 100% | 97.7% | 8.1% |
| 0.75 g-1 g-1 g post-HD | 100% | 97.1% | 100% | 97.9% | 100% | 99.8% | 40.7% |
| 0.75 g-0.75 g-2 g post-HD | **100%** | **97.4%** | **100%** | **98.0%** | **100%** | **100%** | **91.6%** |
| ***Avibactam*** | | | | | | | |
| **Dose** | **Mon** | **Tue** | **Wed** | **Thu** | **Fri** | **Sat** | **Sun** |
| 0.19 g daily | **100%** | **100%** | **100%** | **100%** | **100%** | **100%** | **100%** |
| 0.19 g post-HD | 100% | 93.2% | 100% | 93.4% | 100% | 93.4% | 53.0% |
| 0.19 g-0.25 g-0.25 g post-HD | 100% | 92.7% | 100% | 95.1% | 100% | 95.1% | 64.6% |
| 0.19 g-0.19 g-0.5 g post-HD | 100% | 93.0% | 100% | 93.2% | 100% | 98.2% | 81.9% |

*Gray columns indicate then days when hemodialysis is scheduled; **Bold** dosing regimens are those attaining 90% probability of target attainment on all simulation days with the targets of ≥60%*f*T>MIC of 8 mg/L for cefepime, ≥40% fT>MIC of 2 mg/L for meropenem, ≥50% *f*T>MIC of 8 mg/L for ceftazidime, and ≥50% *f*T>threshold of 1 mg/L for avibactam.
